# Birth weight and gestational age distributions in a rural Kenyan population

**DOI:** 10.1186/s12887-023-03925-2

**Published:** 2023-03-08

**Authors:** Sherri Bucher, Kayla Nowak, Kevin Otieno, Constance Tenge, Irene Marete, Faith Rutto, Millsort Kemboi, Emmah Achieng, Osayame A. Ekhaguere, Paul Nyongesa, Fabian O. Esamai, Edward A. Liechty

**Affiliations:** 1 School of Medicine, Indiana University, Indianapolis, IN USA; 2 Department of Pediatrics, Division of Neonatal-Perinatal Medicine, Indianapolis, IN USA; 3 RTI International, 3040 E. Cornwallis Road, Research Triangle Park, Durham, NC USA; 4 Department of Child Health and Paediatrics, Moi University School of Medicine, Eldoret, Kenya

**Keywords:** Birthweight, Sub-Saharan Africa, Kenya, Gestational age, Obstetrical ultrasound, Fetal growth

## Abstract

**Background:**

With the increased availability of access to prenatal ultrasound in low/middle-income countries, there is opportunity to better characterize the association between fetal growth and birth weight across global settings. This is important, as fetal growth curves and birthweight charts are often used as proxy health indicators. As part of a randomized control trial, in which ultrasonography was utilized to establish accurate gestational age of pregnancies, we explored the association between gestational age and birthweight among a cohort in Western Kenya, then compared our results to data reported by the INTERGROWTH-21st study.

**Methods:**

This study was conducted in 8 geographical clusters across 3 counties in Western Kenya. Eligible subjects were nulliparous women carrying singleton pregnancies. An early ultrasound was performed between 6 + 0/7 and 13 + 6/7 weeks gestational age. At birth, infants were weighed on platform scales provided either by the study team (community births), or the Government of Kenya (public health facilities). The 10^th^, 25^th^, median, 75^th^, and 90^th^ BW percentiles for 36 to 42 weeks gestation were determined; resulting percentile points were plotted, and curves determined using a cubic spline technique. A signed rank test was used to quantify the comparison of the percentiles generated in the rural Kenyan sample with those of the INTERGROWTH-21st study.

**Results:**

A total of 1291 infants (of 1408 pregnant women randomized) were included. Ninety-three infants did not have a measured birth weight. The majority of these were due to miscarriage (*n* = 49) or stillbirth (*n* = 27). No significant differences were found between subjects who were lost to follow-up. Signed rank comparisons of the observed median of the Western Kenya data at 10^th^, 50^th^, and 90^th^ birthweight percentiles, as compared to medians reported in the INTERGROWTH-21st distributions, revealed close alignment between the two datasets, with significant differences at 36 and 37 weeks. Limitations of the current study include small sample size, and detection of potential digit preference bias.

**Conclusions:**

A comparison of birthweight percentiles by gestational age estimation, among a sample of infants from rural Kenya, revealed slight differences as compared to those from the global population (INTERGROWTH-21^st^).

**Trial registration:**

This is a single site sub-study of data collected in conjunction with the Aspirin Supplementation for Pregnancy Indicated Risk Reduction In Nulliparas (ASPIRIN) Trial, which is listed at ClinicalTrials.gov, NCT02409680 (07/04/2015).

## Background

Pediatricians, obstetricians, and public health workers have become accustomed to using fetal growth curves to assess risk for perinatal morbidity and mortality. The widespread use of obstetrical ultrasound early in gestation, particularly within high-income settings, has allowed very accurate and precise estimation of gestational age [1, 2]. Coupled with equally accurate and precise measurements of birth weights, investigators have been able to construct highly accurate fetal growth curves, such as those by WHO [3], Fenton et al. [4], and INTERGROWTH-21^st^ [5, 6].

While early prenatal ultrasound is common in high resource countries, it is less common or even nonexistent in low resource settings. This has made the development of fetal growth curves difficult in these settings. Growing access to low-cost ultrasound (US) devices in these settings may begin to increase access to antenatal sonography for populations in low-to-middle income countries [7, 8]. Fetal growth curves are used to categorize infant gestational age and quality of intrauterine growth, which is vitally important for clinical care of the newborn, as well as for evaluating the impact of public health programs and pregnancy interventions, such as those related to maternal nutrition [9]. However, one challenge is that one cannot merely assume the growth curves or birthweight charts in one global region may be applicable in another region. For example, there are well known differences in birth weight distributions between Africa and Asia [10]. There is some evidence that customizing fetal growth curves for particular populations may result in higher sensitivity and specificity to determine small-for-gestational and low birthweight neonatal outcomes, as compared to using broad based population charts across more heterogenous regions [11]. Detecting these differences via use of ultrasound data, as compared to other methods, such as fundal height, improves accuracy, particularly in regards to stratifying risk for neonatal mortality [12, 13]. Therefore, it is crucial that each global region has population specific fetal growth curves for accurate classification of infants by birth weight within that region.

The Global Network for Women’s and Children’s Health Research recently completed a multi-site global clinical trial of low dose aspirin administered to women throughout pregnancy, beginning between 6–14 weeks gestation [14]. To assure women met the gestational age requirements for the study, all participants received an ultrasound prior to study entry. Therefore, a cohort of women resulted who had all undergone gestational age assessment prior to 14 weeks of pregnancy. Women were followed throughout pregnancy, and birthweights of resulting infants obtained as soon as possible after birth. With this cohort of accurate gestational age and birthweight data, we have generated estimated fetal growth curves for the Kenya site. There is evidence that customized birthweight cohort data may be more accurate for detecting fetal growth restriction related to placental dysfunction – an important hypothesized contributor to premature birth – as compared to utilizing population-based birthweight information. [15]

One of the largest, most comprehensive set of studies on multinational birth weight are those of the INTERGROWTH-21st trial [16, 17]. These studies were specifically designed to generate accurate, longitudinal, and multinational fetal and childhood anthropometric measurements. INTERGROWTH-21st subjects were recruited from eight countries, including from the environs of Nairobi, Kenya.

## Methods

**Fig. 1:**
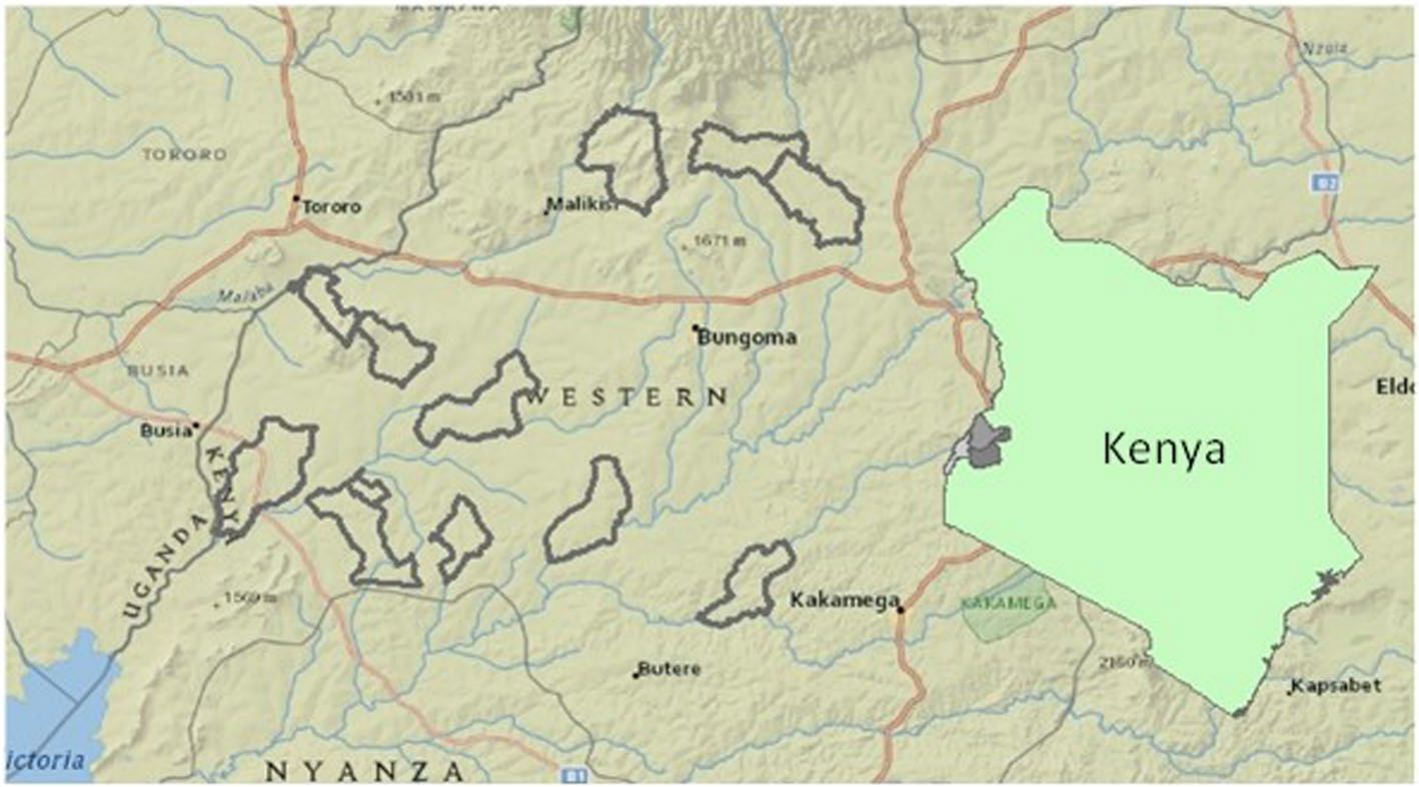
Map of the study region, located in Busia, Bungoma, and Kakamega counties of western Kenya. Study clusters are outlined in gray. County locations within Kenya are depicted in the inset map

The data presented in this paper were acquired at the Kenya site as part of the Global Network for Women’s and Children’s Health Research ASPIRIN trial. Detailed study methods are described in Hoffman et al. [14, 18]. The Kenyan site (Fig. 1) is situated within the malaria holoendemic Lake region of Western Kenya, specifically the counties of Busia, Kakamega, and Bungoma [19]. The eight geographical clusters within the Kenyan site are served by over 20 health facilities, most operated by the government and staffed by nurse-midwives, clinical officers, and a single medical officer. Three hospitals in the area function as county referral hospitals [20]. There is one tertiary teaching and referral hospital based in Eldoret for the western region with a newly established training program in maternal fetal medicine. Most physicians are generalists, with some trained obstetricians and pediatricians [20].

Eligible subjects were pregnant nulliparous women carrying singleton pregnancies. An early ultrasound was performed between 6 + 0/7 and 13 + 6/7 weeks gestational age for accurate pregnancy dating. From this ultrasound, the estimated day of delivery was determined using the ACOG algorithm [21, 22], which was programed onto a handheld android device. Eligible women were then randomized to a daily regimen of low dose aspirin or placebo and followed to 42 days post pregnancy completion. Randomization was performed by site, with the randomization sequence for each site provided by the data coordinating center (RTI) using a computer algorithm based on a randomly permuted block design with varied block sizes. The primary analysis included data from Kenya and 5 other countries (India, Pakistan, Guatemala, Zambia, and Democratic Republic of Congo) and found that the aspirin intervention reduced delivery < 34 weeks; no impact on birthweight was observed [14]. Within the Kenya population, mean birthweight and gestational age were comparable by treatment arm. The analysis of variance was statistically significant for birthweight (*p* = 0.0167), but the difference of means was not clinically significant (~ 63 g). The analysis of variance for gestational age was not statistically or clinically significant.

Infants born to subjects were weighed on platform scales either at a delivery in a health facility, or if born outside of a facility, at the home of the local village elder [23]. For infants delivered at participating public health facilities, the weighing scales used were those provided by the Government of Kenya; our study team did not have control over the make or model of infant weighing scales utilized. The weights of infants born within the community-setting, and weighed by village elders, were obtained using scales (Perlong Medical Equipment Co., Ltd.RGZ-20 Nanjing, China) provided by our research team. Only infants with a measured birth weight (BW) were included in this analysis.

For subjects experiencing either a stillbirth or an infant death before the 42-day follow-up period, the assumed cause of death was determined using a previously published algorithm [24, 25]. Estimated gestational age (EGA) in days at time of delivery or stillbirth was defined as (Date of delivery – Estimated Date of Delivery by ultrasound) + 280. EGA in weeks was defined as EGA Days/7. Completed weeks of gestation was calculated by rounding the EGA weeks to the next larger integer.

Statistical analyses were performed using JMP software and SAS version 9.4 (SAS Inc, Cary, NC USA). The 10^th^, 25^th^, median, 75^th^, and 90^th^ BW percentiles for 36 and 43 completed weeks gestation were determined, the resulting percentile points were plotted, and curves determined using a cubic spline technique. Percentile curves for gestational ages less than 36 weeks (*n* = 57) or greater than 43 weeks (*n* = 9) were not plotted due to paucity of data for these groups.

A signed rank test was used to quantify the comparison of the percentiles generated in this study with those of the INTERGROWTH-21st study. This test was performed twice within each gestational week – once testing the null hypothesis that the Kenya Male median equals the reported median of the INTERGROWTH-21st Male data, and once testing the null hypothesis that the Kenya Female median equals the reported median of the INTERGROWTH-21st Female data. This analysis was non-directional and performed at the alpha = 0.05 significance level.

## Results

**Fig. 2:**
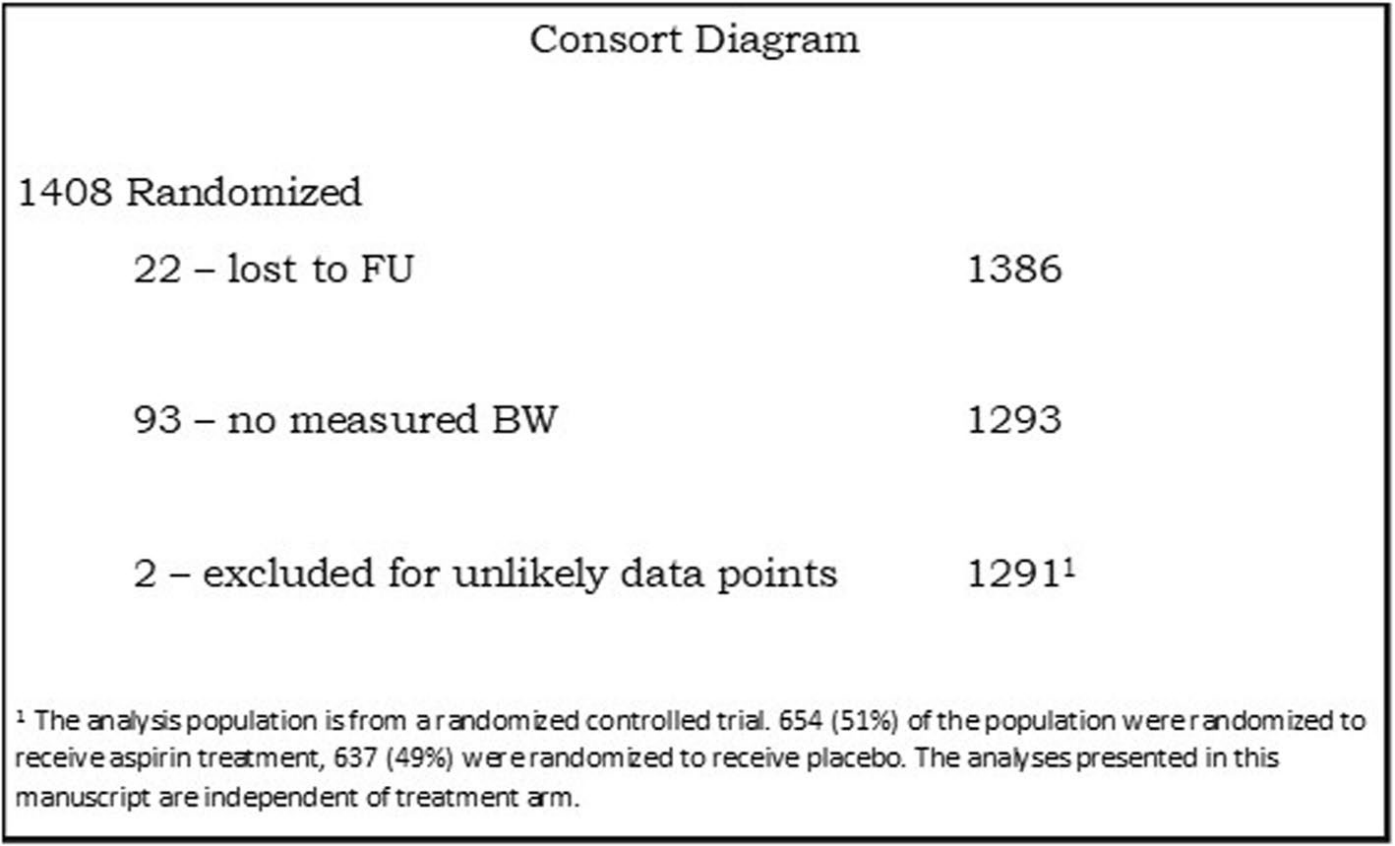
Consort diagram

The consort diagram is shown in Fig. 2; 1408 women were randomized. Twenty-two did not have delivery gestational age data and are considered lost to follow-up (LTFU), and 93 infants did not have a measured birth weight. Miscarriages accounted for the majority (49) of the subjects with missing measured BW. Twenty-seven stillbirths, 5 infant deaths, and 12 survivors lacked measured birth weights. Two additional infants were excluded because of highly unlikely birth weight/gestational age combinations. Therefore, 1291 infants were included in the final data set. However, for the estimation of birthweight by gestational age percentiles, data were restricted to gestational ages 36–42 weeks, due to small numbers outside these parameters, as well as to compare with the INTEGROWTH21 data. This resulted in *n* = 1189.

Table 1 shows maternal age and gestational age at study entry for each excluded group and the final analysis group. No significant differences were found between subjects who were lost-to-follow up for measured birth weight, or included in the final data set.

**Table 1:** Maternal age (years) and gestational age (weeks) at study entry

	All (1408) Mean	SD	LTFU (22) Mean	SD	No BW (93) Mean	SD	BW (1291) mean	SD	p
Maternal Age	20.1	3.0	19.8	2.8	19.7	3.0	20.1	3.0	NS*
GA at Entry	10.4	2.1	10.9	2.2	10.2	2.1	10.4	2.1	NS*

Note: Maternal age (years) and gestational age (weeks at study entry), for all randomized, those lost to follow-up, those with no measured birth weight, and those with a measured birth weight; these constituted the final analysis group. N for each group is in parentheses

^*^NS *p* < 0.05

The majority of subjects (*n* = 1081) delivered in a health facility, while 210 delivered at home. Deliveries were conducted primarily by nurse-midwives (1103); 106 deliveries were conducted by Traditional Birth Attendants, 33 by either self or a family member, and 49 by a physician. The vast majority of birth weights were obtained on day of life (DOL) 0 (91%); 98% were obtained within DOL 0–3.

**Fig. 3:**
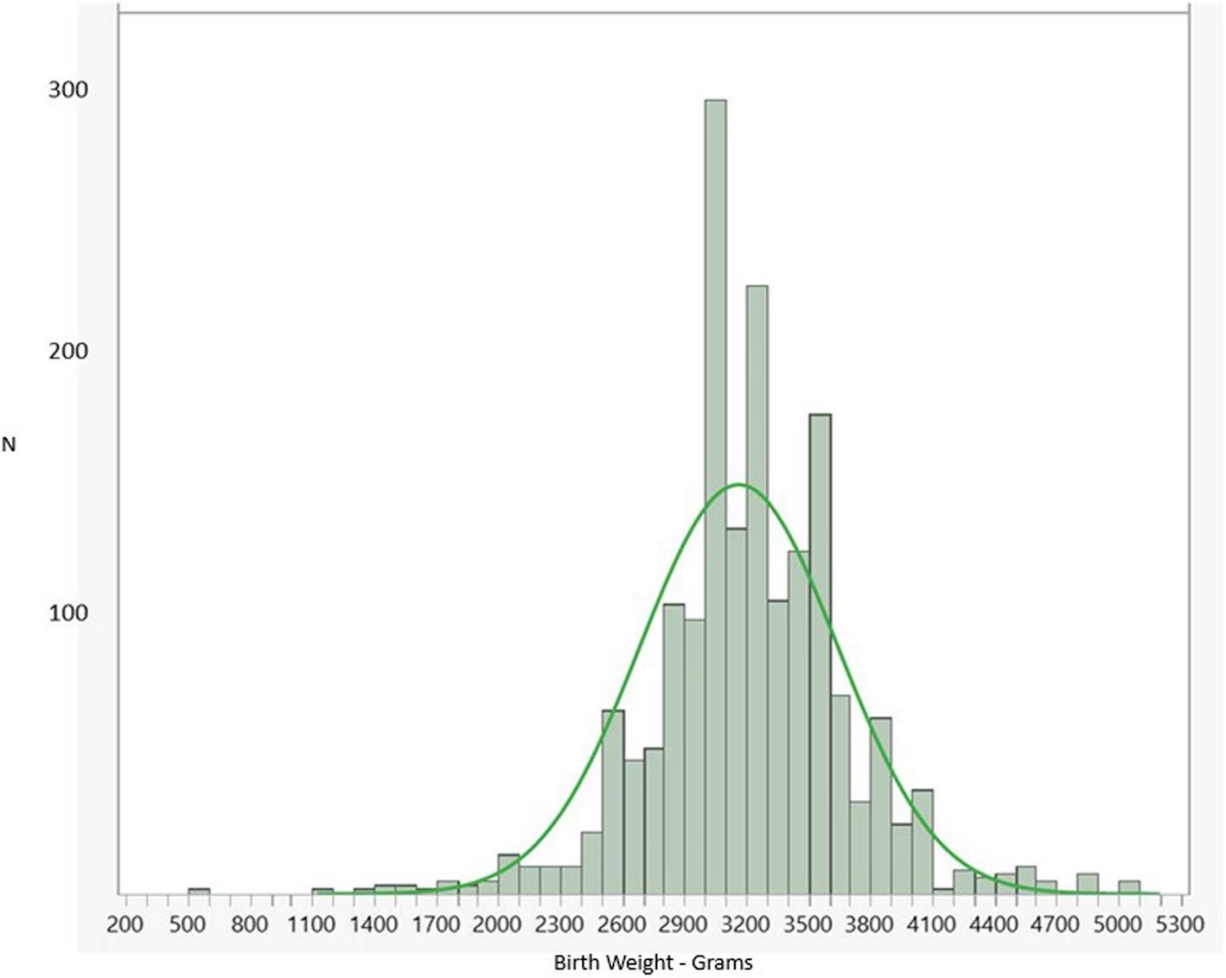
Histogram of measured birth weights. A fitted normal distribution curve is projected onto the actual data

Figure 3 shows the birth weight distribution. A normal distribution model is projected onto the histogram. The histogram shows evidence of digit preference in the recording of birth weights, which affected the Anderson–Darling Goodness of Fit of the normal distribution model (< 0.001). It is also possible that the normal distribution model was rejected due to the limited precision of the data, resulting in a large number of tied data points [26].

**Fig. 4:**
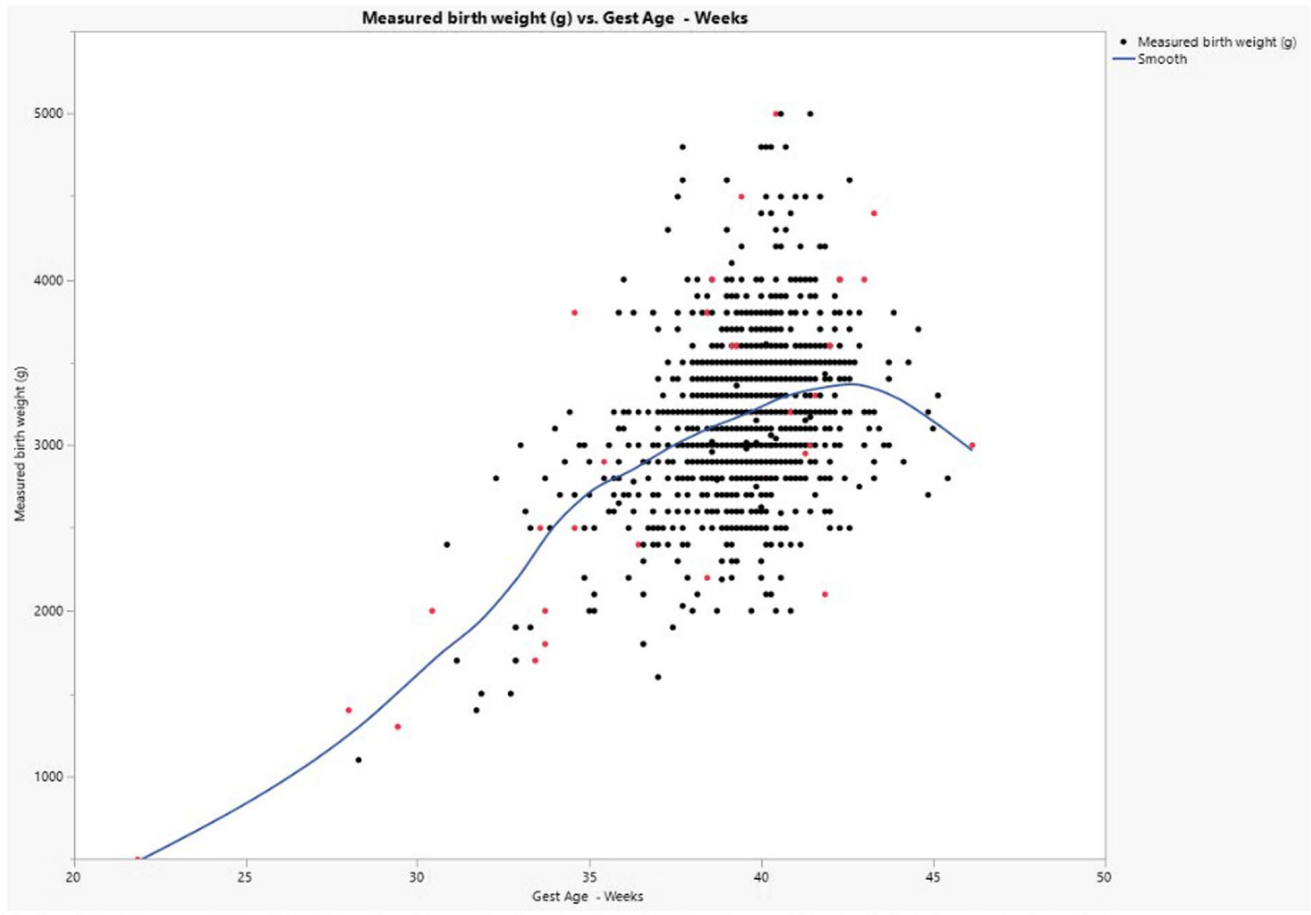
Scatterplot of Measured Birth Weight (y axis) and gestational age (x axis). A cubic spline was fitted and plotted onto the data. Red points indicate either a fetal or neonatal death

Figure 4 depicts a scatterplot of gestational age and measured birth weight, with a cubic spline line fitted to the data. Deaths (*n* = 32, 2.5%), either stillbirth or early infant death (0–6 days postnatal age), are plotted in red, whereas survivors are plotted in black. Causes of death for the live born infants (*n* = 19) were primarily asphyxia (*n* = 10) followed by prematurity (*n* = 3), infection (*n* = 4), congenital anomalies (*n* = 1) and unknown (*n* = 1). Stillbirths (*n* = 13) were primarily due to asphyxia (*n* = 10), followed by infection (*n* = 2) and unknown (*n* = 1).

**Fig. 5:**
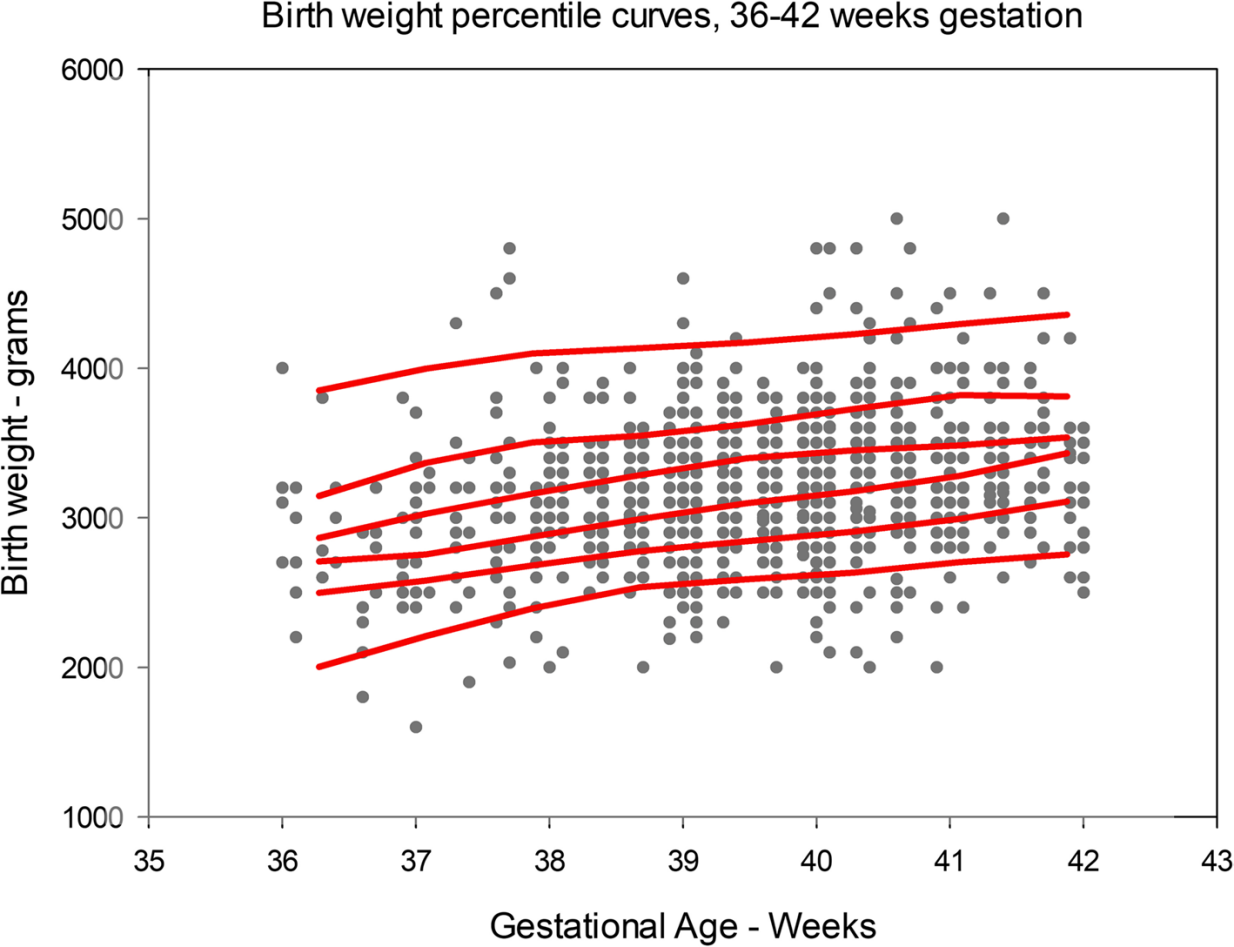
Birth weight percentile curves are plotted against actual data (*n*=1189). Only percentiles for 36—42 weeks are plotted due to paucity of data in the other completed weeks of gestation, as well as facillitating comparison with INTERGROWTH 21 data

Excluding stillborn and early neonatal death infants, the 10^th^, 25^th^, 50^th^, 75^th^, and 90^th^ birth weight percentiles for 36–42 completed weeks of gestation are shown in Table 2. Percentiles for completed weeks of gestation less than 36 or greater than 42 have been excluded due to paucity of data in each of these gestational week categories. The data points for these percentiles are plotted as curves onto the birth weight:gestational age actual data in Fig. 5.

**Table 2:** Birth weight (g) percentiles by completed weeks of gestation, 36 – 42 weeks gestation

Gestational age	n	p10	p25	median	p75	p90
36	34	2300	2500	2740	3100	3200
37	82	2400	2800	3000	3200	3500
38	199	2700	2900	3000	3300	3500
39	352	2700	3000	3200	3400	3700
40	310	2800	3000	3200	3500	3800
41	158	2900	3000	3300	3500	4000
42	54	2800	3200	3450	3600	3800

Table 3 shows the 10^th^, 50^th^, and 90^th^ percentiles for birth weight for the present study and for the INTERGROWTH-21st study; *p*-values for the signed rank test comparison of medians are also reported in this table. To ensure comparability with the INTERGROWTH-21^st^ data, this comparison uses a subset (*n* = 1189) of the Kenya population, excluding stillbirths, early infant deaths, and gestational ages < 36 or > 42. Births at 33–35 weeks and 43 weeks gestations were excluded due to paucity in data. Furthermore, due to limited public data for INTERGROWTH-21^st^, the analysis was limited to single data-point (median) comparisons, which does not address the intricacies of the full fetal growth curve distribution. Although this analysis had limitations the observed significant differences warrant further research with more robust data and methods in the rural, Western Kenyan population.

**Table 3:** Birthweight Percentiles by Gestational Age for Rural Kenya and INTERGROWTH-21st

		**Kenya**				**INTERGROWTH-21st Overall **[5]				
**Sex**	**Week**	**n**	**10th**	**50th**	**90th**	**n**	**10th**	**50th**	**90th**	***p*-value**
Males	36	18	2200	2740	3800	323	2180	2690	3250	0.3396
	37	45	2300	2900	3500	857	2380	2890	3450	0.8808
	38	108	2700	3100	3500	2045	2570	3070	3630	0.5198
	39	177	2700	3200	3700	3009	2730	3240	3790	0.5313
	40	159	2800	3200	3900	2568	2880	3380	3940	0.0000
	41	80	2950	3300	3950	1179	3010	3510	4060	**0.0001**
	42	34	2900	3500	3800	206	3120	3620	4170	**0.0017**
Females	36	16	2300	2800	3200	293	2140	2600	3120	**0.0407**
	37	37	2700	3100	3700	803	2330	2800	3320	0.0000
	38	91	2800	3000	3500	1802	2500	2970	3510	**0.0001**
	39	175	2600	3100	3700	2869	2650	3130	3660	0.7835
	40	151	2800	3300	3800	2523	2780	3260	3800	0.7912
	41	78	2800	3200	4000	1195	2890	3370	3920	0.0867
	42	20	2675	3350	3750	224	2980	3460	4010	0.0807

Weeks are completed weeks of gestation. INTERGROWTH-21st data have been converted from kg to grams and reported *p*-values represent a signed rank comparison of whether the observed median of the rural Kenya data differs from a hypothesized median value based on the medians reported in the INTERGROWTH-21st distributions (significant values in bold)

## Discussion

Our data complements that reported by INTERGROWTH-21st, in that our Kenyan subjects were recruited from a rural agricultural setting with a significant malaria burden, whereas the Kenyan subjects in the INTERGROWTH-21st trial were recruited from the Parklands area of suburban Nairobi. Parklands is considered a middle to upper socioeconomic urban area, while the area of our study is largely defined as a World Bank rural poverty area. Thus, our population demographics are considerably different than those of the Kenyan subjects included in the INTERGROWTH-21st study. Despite these in-country geographical variations, our results, from rural Western Kenya, are quite similar to those reported for INTERGROWTH-21^st^.

The incidence of prematurity and low birth weight are important public health indicators for a population. In addition, the construction of birth weight percentiles for gestational age are crucial for establishing the intrauterine growth of an infant, which is then used to track postnatal growth. Abnormal postnatal growth, as evidenced by deviations from the expected weight for age percentile that are established by the assessment of birth weight for gestational age, is then used as a warning sign for the need for nutritional intervention. However, it is known that the birth weight distribution curve varies among populations. In addition, despite extensive study, it has been challenging for clinicians and public health practitioners to find accurate prenatal surrogate markers that correlate reliably with actual birthweight [13, 27, 28]. Reasons may include genetics, environmental factors, and average maternal nutritional status. As evidenced by the Dutch famine in World War II, birth weight can clearly be adversely affected by the general population nutrition level and access to adequate food [29, 30].

Birth weight and gestational age are intertwined, and it is crucial in evaluating a population to be able to separate low birth weight due to prematurity and low birth weight due to fetal factors, either constitutional or nutritional. Therefore, fetal growth curves with accurate birth weights are important tools for evaluating public health interventions designed to reduce rates of stillbirth [31, 32], premature birth, low birthweight, and neonatal mortality [33]. However, accurate and precise estimation of gestational age is difficult, and generally only attained either in an in vitro fertilization or by means of an early (less than 14 weeks) fetal ultrasound capable of making accurate anthropometric measurements. While these ultrasounds have become commonplace in high resource countries, they are less common or completely inaccessible in low resource settings, where global rates of neonatal morbidity and mortality are highest, and where increased exposure to infectious and non-infectious diseases [34, 35], deleterious environmental exposures [36], poor access to early and comprehensive antenatal care [37, 38], and/or sub-optimal maternal nutritional factors [39–41] are likely to have the greatest impact on fetal growth. The data presented in this study represent pregnancies for which all acquired gestational dating, via obstetric ultrasonography, at less than 14 weeks.

The INTERGROWTH-21st study found that for most gestational weeks, median birthweights differed for male and female infants. The signed rank test used for this analysis was performed separately for male and female data but was limited by sample size disparities. Although this analysis had limitations and was based on a single metric (median), the observed significant differences at lower gestational ages for females and higher gestational ages for males warrant further investigation in the rural, western Kenyan population. A major strength of our cohort is the data originated from a prospectively designed study. Subjects were enrolled early in pregnancy as part of the ASPIRIN trial, and tracked prospectively through delivery to 42 days postpartum, as part of our established, population-based maternal newborn health registry, which employs rigorous quality assurance procedures [42]. This design ensured that, as much as possible, all pregnancies were tracked, not just those enrolling late in gestation or even after delivery. In retrospective studies, stillbirths and miscarriages are the most likely type of fetal/early neonatal deaths to be missed, thereby skewing the data towards a heavier birthweight distribution. Therefore, we elected to include all stillbirths and early neonatal deaths for whom accurate measured birth weights were obtained (Fig. 4). However, we did exclude these data from the percentile calculations, as these were also excluded in the INTERGROWTH-21st data. The ASPIRIN trial showed no differences in birth weight between the active drug and placebo groups [14]. For this paper, we performed a subgroup analysis of the Kenyan subjects, and a small (~ 60 g) difference in the mean birth weights was found. We believe this small difference to be clinically insignificant, and therefore did not stratify the analyses by active drug or placebo.

In the present study, another strength was our very low loss-to-follow up rate; we lost only 22 subjects (1.56%) to follow-up before birth. Another key strength is the rigorous quality-assurance standards that were utilized to train, monitor, and evaluate the sonographers in this study [43, 44]. The ultrasound sonography devices that we utilized (GE LOGIQ e systems using wide-band (2.0–5.5 MHz) convex array transducers (GE Healthcare, Milwaukee, WI) are of high quality, providing additional confidence in regards to the accuracy of fetal growth measurements that were obtained among our cohort.

There are several limitations of our study. First, the sample size is relatively small, especially for infants born below 36 weeks and of less than 2500 g. Furthermore, we used data only from women who had qualified for and enrolled in the ASPIRIN study, as these women all had accurate ultrasound gestational dating. This limited our subjects to primagravidas, and to singleton pregnancies, consistent with the ASPIRIN eligibility requirements. It is well established that multiple gestation pregnancies are more likely to result in low birthweight infants. Birth spacing can also impact the birthweights of subsequently born sibling infants, as compared to first born infants [45].

Similar to challenges faced by other groups (e.g., EN-INDEPTH; Blencowe, et al., 2021) [46] another significant limitation in our study is the lack of birth weight data for many stillbirths and early neonatal deaths. In general, it can be difficult, within the sub-Saharan setting, to obtain accurate birthweight data for very low birthweight infants (e.g., below 1500 g). This is especially problematic for estimating mortality risk of infants less than these parameters. We made strenuous efforts to obtain these data, but cultural practices and a deep stigma related to stillbirth limited our ability to do so [47, 48]. It is the cultural practice in our study population to immediately inter stillbirths and neonatal deaths, making it nearly impossible to obtain birth weight data on this subset of the population [48]. Depending on the actual birth weights of these subjects, however, it is possible that their loss did skew our data.

As mentioned previously, we had a very low loss-to-follow-up rate, overall. However, an additional 93 subjects, while not lost to follow-up, did not have measured birth weights. Of these, 49 pregnancies ended before 20 weeks and were considered miscarriages. Of the remaining subjects, 27 were stillbirths, 5 were neonatal deaths, and 12 had unknown status. A final challenge faced in this study is the limited precision of the scales used to determine birth weights. In general, these scales were graduated at 50-g increments. Combined with documented “digit preference” bias from other settings, including within the East African region [49, 50], and for which there might be some anecdotal evidence within our Network [10], this lack of granularity in regards to birthweight measurements limits the interpretation of our results.

## Conclusions

Premature birth continues to be a major problem in sub-Saharan Africa, including Kenya. The comparison of our data, with INTERGROWTH-21st results, found preliminary signals that this rural-dwelling population may have birthweight by gestational age percentiles that differ from that currently reported in global data sources. These results further indicate that continued efforts by clinicians and public health practitioners are needed to develop timely, accurate, effective, acceptable, and feasible evidence-based methods to accurately detect and predict regionally-specific rates of fetal growth restriction and low birthweight [15, 51, 52]. Future analysis of rural Kenyan fetal growth curves in relation to perinatal mortality risks and confounding risk factors is warranted. Within low/middle-income settings, this will be an important contribution toward on-going global efforts to reduce overall rates of neonatal mortality [53, 54].

## Data Availability

The datasets used and/or analysed during the current study are available from the corresponding author on reasonable request.

## Abbreviations

ACOG: American College of Obstetricians and Gynecologists
ASPIRIN Trial: Aspirin Supplementation for Pregnancy Indicated Risk Reduction In Nulliparas
BW: Birthweight
DOL: Day of life
EGA: Estimated gestational age
EN-INDEPTH: Every Newborn-International Network for the Demographic Evaluation of Populations and their Health
GA: Gestational age
INTERGROWTH-21^st^: The International Fetal and Newborn Growth Consortium for the 21st Century
